# Estimation of Gross Motor Functions in Children with Cerebral Palsy Using Zebris FDM-T Treadmill

**DOI:** 10.3390/jcm11040954

**Published:** 2022-02-12

**Authors:** Mariusz Bedla, Paweł Pięta, Daniel Kaczmarski, Stanisław Deniziak

**Affiliations:** Faculty of Electrical Engineering, Automatic Control and Computer Science, Kielce University of Technology, al. Tysiąclecia Państwa Polskiego 7, 25-314 Kielce, Poland; p.pieta@tu.kielce.pl (P.P.); d.kaczmarski@tu.kielce.pl (D.K.); deniziak@tu.kielce.pl (S.D.)

**Keywords:** Gross Motor Function Measure, cerebral palsy, estimation, Zebris FDM-T, regression

## Abstract

A standardized observational instrument designed to measure change in gross motor function over time in children with cerebral palsy is the Gross Motor Function Measure (GMFM). The process of evaluating a value for the GMFM index can be time consuming. It typically takes 45 to 60 min for the patient to complete all tasks, sometimes in two or more sessions. The diagnostic procedure requires trained and specialized therapists. The paper presents the estimation of the GMFM measure for patients with cerebral palsy based on the results of the Zebris FDM-T treadmill. For this purpose, the regression analysis was used. Estimations based on the Generalized Linear Regression were assessed using different error metrics. The results obtained showed that the GMFM score can be estimated with acceptable accuracy. Because the Zebris FDM-T is a widely used device in gait rehabilitation, our method has the potential to be widely adopted for objective diagnostics of children with cerebral palsy.

## 1. Introduction

Cerebral palsy (CP) is a group of permanent disorders of the development of movement and posture that are considered one of the most frequent causes of non-progressive motor disability in children [1,2]. Symptoms of CP vary from person to person, ranging from mild to very severe movement difficulties [1,2]. CP is a very diverse problem accompanied by multiple comorbidities such as communication impairment, cognitive impairment, visual and hearing impairment, reduced alertness, epilepsy, seizures, musculoskeletal problems, feeding difficulties, behavioral disorders, mental retardation, and even sleep disorders [1,2,3,4,5]. Due to the complexity of this condition and the fact that there is no cure for CP, its treatment is also very complex: it includes botulinum toxin therapies, certain surgical techniques (such as orthopedic surgery and rhizotomy), and supportive treatments (such as physiotherapy or focal vibration (FV) on limb muscles)—these procedures help alleviate symptoms of the disease and improve motor skills of patients [1,6].

The volume of data that describe patients with CP is constantly expanding, and the diversity of these data is also increasing [3,4,5,7,8,9]. A significant problem associated with CP is the dispersion and inconsistency of data related to this condition, as well as the lack of dedicated information systems that would facilitate the observation of patients and help determine the most appropriate treatment process [2]. The Mobilize Center information system addresses the problem of data spread [10]. Moreover, in this work Ku et al. identified Big Data Analytics (BDA) and Machine Learning (ML) as potential methodologies that could revolutionize research on human mobility. Over the last few years, these approaches have been used extensively in numerous studies related to CP [11,12,13,14,15,16,17,18,19,20]. The growing amount of data obtained as a result of human biomechanical studies makes it imperative to develop advanced methods of multivariate analysis and ML. Current trends and future directions regarding Computer Vision (CV) and ML in CP research have been summarized in several works [9,21,22].

Because CP is a complex disorder, different morphological and functional Gait Classification Systems (GCSs) have been developed, many of whom express either gait pathology or functional impairment [23,24]. GCSs help categorize gait pathologies, allow for the assessment of patient health over time, provide a standardized way to compare gaits between different patients, simplify treatment recommendations and planning, and improve data exchange between clinicians and researchers [23,24]. To increase the efficiency of gait classification and quantify gait compared to the unimpaired gait of typically developing individuals (TD), data obtained with the use of three-dimensional Instrumented Gait Analysis (3DIGA) have been used to introduce gait indices such as the Gillette Gait Index (GGI), the Gait Deviation Index (GDI), and the Gait Profile Score (GPS) [24,25,26]. GGI has been proven to show an excellent correlation with Gross Motor Function Measure (GMFM), and GDI is strongly correlated with Gross Motor Function Classification System (GMFCS) [24]. Also, it should be noted that GDI and GPS were not developed exclusively for gait assessment in patients with CP, but as a more general measure of gait pathology [24,26]. Another test that is frequently used to evaluate functional exercise capacity of children with CP and is inexpensive to administer is the 6-minute Walk Test (6MWT) [27,28]. Furthermore, a work by Thomason et al. proposed a novel assessment of gait function in children with CP—the Gait Outcomes Assessment List (GOAL) [29]. This index tries to capture the complex nature of physical ability by taking into account contextual factors that contribute to functioning, as well as expectations of children and their parents.

Some of the GCSs are more efficient to administer, such as GGI and GDI, but require sophisticated and expensive equipment to measure certain kinematic and spatiotemporal parameters of a patient’s gait [24]. Other indices like GMFM do not require the use of such equipment, but have other disadvantages. Because the GMFM score is evaluated using 88 (GMFM-88) or 66 (GMFM-66) items (measurements) grouped into 5 dimensions, the process of obtaining a value of this index can be time consuming—typically, it takes 45 to 60 min for the patient to complete all tasks, sometimes in two or more sessions [30,31]. Individual test items are scored from 0 to 3, and even though trained therapists reach a high level of agreement when administering and scoring GMFM [30,31], it may not be the case for young, inexperienced, untrained therapists, or those unfamiliar with the method of measurement. Moreover, if the child is tired or other factors related to the child are present, it is not always feasible to complete all the measurements [31]. To increase the efficiency of the evaluation of the GMFM index, its abbreviated forms were developed: GMFM-66-IS and GMFM-66 B&C [32]. GMFM-66-IS can be administered in approximately 20 to 30 min [31], so the time needed to evaluate the GMFM score can be reduced by two-thirds. A recent work proposed another reduced version of the GMFM-66 index named rGMFM-66 [31]. In this study Duran et al. used several artificial intelligence approaches to estimate the GMFM-66 value with the fewest possible measurements, e.g., by the means of Random Forest (RF), or Support Vector Machine (SVM).

An important direction of research on CP is the search for associations in patients health data, e.g., to find correlations with various classification systems and measures, such as: the Communication Function Classification System (CFCS) [33,34], the Five-Times-Sit-to-Stand Test (FTSST) [35], GDI [35,36,37,38,39,40], GMFCS [33,34,36,41,42], GMFM [43], the Manual Ability Classification System (MACS) [34], and also to identify other associations and correlations [39,40,44,45,46,47,48,49,50]. Strongly correlated data are essential for BDA and ML algorithms, e.g., to develop successful regression models.

Another direction of research is an attempt to predict the values of some of the previously mentioned classification systems and measures, as well as other health parameters of patients with CP, mainly by means of ML. In Ries et al. [51] developed a statistical orthosis selection model using RF. The goal of this model was to predict which of the five orthosis designs would provide the best gait outcome (defined as the change in GDI) for patients with diplegic CP. In Galarraga et al. [52] predicted postoperative lower limb kinematics utilizing multiple linear regressions. Another study by these authors [53] proposed a system that predicts postoperative kinematics considering a large number of surgical combinations and gait patterns in CP. In [54], Rosenberg and Steele used musculoskeletal models to simulate walking kinematics of children with CP, with and without passive ankle foot orthoses (AFOs). The work of Rajagopal et al. [55] built regression models to estimate the effect of single-event multilevel surgery (SEMLS) in improving gait in patients with CP. In [56], Duran et al. developed a method that predicts the expected changes in the GMFM-66 measure of individual children with CP between two points in time that are 6 months apart. Pitto et al. in their research [57] developed the SimCP—a novel framework that allows to evaluate the outcome of different simulated surgeries, enabling clinicians to predict gait performance after orthopedic intervention in children with CP. In Kidziński et al. [58], presented ML models to predict clinically relevant motion parameters such as walking speed, cadence, knee flexion angle at maximum extension, and GDI. The methods used by the researchers included the Convolutional Neural Network (CNN), RF, and Ridge Regression (RR)—they were trained using ordinary videos of patients with CP. Another similar work was conducted by Jalata et al. [59]. They proposed a Graph Convolutional Neural Network (GCNN) that predicts similar gait measures based on a video of a patient. The research by Azhand et al. [60] demonstrated a novel gait assessment model using CNNs that can extract 3D skeleton joints from videos of walking humans taken with monocular smartphone cameras. Other interesting work by Afifi [61] proposed a model using RF to predict CP in very preterm infants. The model provided a good level of discrimination between children with and without CP.

The purpose of this study was to develop a method to estimate the GMFM measure for patients with cerebral palsy based on the Zebris FDM-T treadmill results. The proposed model was trained using data collected from 23 patients with CP. The Zebris FDM-T is a device that is widely used in gait rehabilitation, so our method has the potential to be widely adopted. Section 2 briefly characterizes the GMFM measure and the Zebris FDM-T treadmill device, as well as discusses the study population and data analysis methods used to carry out this work. Section 3 presents the experimental results obtained with our method. Finally, Section 4 provides the interpretation of these results and concludes the article highlighting future research directions.

## 2. Materials and Methods

The Materials and Methods section has been divided into 5 subsections. The first subsection describes the GMFM, an observational tool for measuring changes in gross motor function over time in children with cerebral palsy. Then, the Zebris FMD-T treadmill device was characterized, from which the results for analysis were obtained. The third subsection covers the study population, which was based on the use of data from 23 patients. The next subsection discusses the Zebris FDM-T treadmill diagnostics scheme that was used to obtain patient measurements. The last subsection describes the data analysis methods that were used to carry out our research.

### 2.1. Gross Motor Function Measure (GMFM)

The GMFM is a precise observational tool for measuring changes in gross motor function over time in children with CP [47]. It is based on the principles of developmental neurophysiology [62,63]. It is a quantitative scale: it was designed to minimize variation by including the assessment of ’does do’ rather than ’can do’ (it consists of objectively defined test items and it has a standardized scoring system) [64]. The GMFM examines the functional behavior of children in terms of major motor activities from infancy to 16 years of age. It focuses on monitoring the number of gross motor activities that a 5-year-old child can perform [32]. It is an insightful and comprehensive tool that is used to assess the effectiveness of various treatments due to its high sensitivity and repeatability [38,62,65]. In addition, it can be utilized to set the functional goals of the current or planned treatment of the child. Also, it allows one to determine the functional state of the child at a given moment and compare it with the previous state. Lastly, it can be used to establish the type of functional process of the child.

There are two versions of GMFM: GMFM-66 and GMFM-88 [66]. The GMFM-88 scale was developed for children with CP, but it is also used in children with Down syndrome and brain injuries [62]. It provides a more detailed description of the limitations and abilities of children with varying levels of motor disabilities [66]. The GMFM-88 can be administered with shoes and outpatient aids and/or orthoses [62]. The GMFM measures a child’s ability in five different dimensions:

1. lying and rolling (17 measurements),
2. sitting (20 measurements),
3. crawling and kneeling (14 measurements),
4. standing (13 measurements),
5. walking, running, and jumping (24 measurements) [32,47,65].

During the GMFM assessment, the child receives points from 0 to 3 for each motor task. The obtained number of points reflects the degree of execution of a given activity:

- 0 points—does not initiate movement,
- 1 point—activity performed in the range below 10% (initiates movement),
- 2 points—activity executed in the range between 10–100%,
- 3 points—activity performed in 100%,
- NT—not tested [66].

A child scores 0 points for omitting a measurement or for being unable to complete one [66]. The points obtained are then calculated by the computer program GMAE (Gross Motor Ability Estimator), which returns the result as a percentage with a 95% confidence area [66]. The GMFM scale is used in rehabilitation centers that treat children with CP, although it cannot be administered to patients with behavioral disorders and those who do not understand verbal commands or demonstrations [66,67]. The tool allows monitoring even the smallest progress in motor development of children with CP, especially those with spastic forms of the disease, which is an important step in improving their functional state [66]. All information about progress in child activity is also of key importance for parents of young patients. It is recommended to complete all test items, even by older children who may be able to perform more advanced tasks. The final report should also contain information in the event of difficulties arising from lack of cooperation with a child [66,67].

### 2.2. Zebris FDM-T Treadmill Device

The Zebris FDM-T treadmill [68] is a very universal rehabilitation and diagnostic device that supports various modes of operation. It can be used both for dynamic gait analysis and for static analysis while a patient is standing. The treadmill is used for the diagnosis and rehabilitation of lower limbs in patients with CP. In addition, a camera can be connected to the device, as well as an electromyography (EMG) set and a projector to display the footprints on a treadmill that a patient should follow, or a monitor that displays a customizable track with obstacles that a patient should avoid. Measurement results can be exported in the form of text, graphics, and video. Basic parameters that can be measured include values of forces and pressures of a feet, as well as time-space parameters of gait [69].

In this study, the results of the following measurements obtained with the use of the Zebris FDM-T device were analyzed: static analysis when standing with eyes open and closed, and gait analysis at a preferred speed determined for each patient at the first attempt. During the stance analysis, more than 20 parameters are recorded, such as forces, COP positions, 95% confidence ellipse area, etc. More than 100 parameters representing different aspects of gait are recorded during gait analysis, such as forces, pressures, gait phases, times, anterior/posterior position, foot rotations, lateral symmetry, cadence, velocity, step length, step width, etc.

### 2.3. Study Population

**Figure 1:**
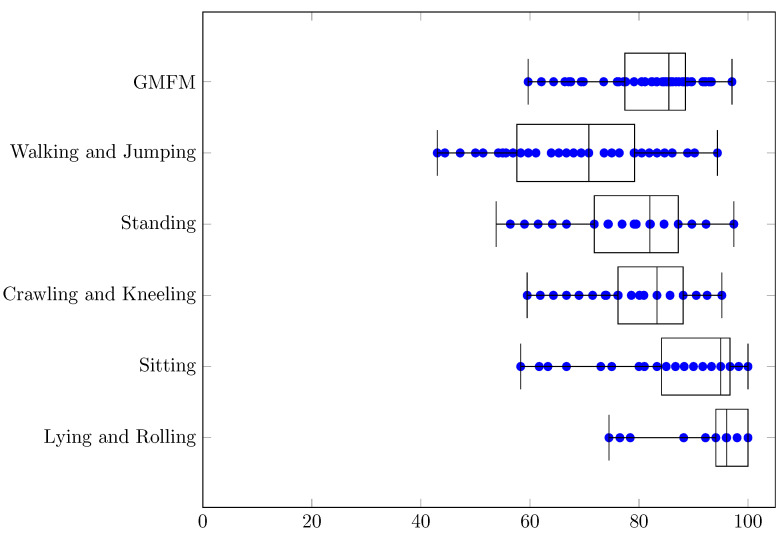
Patients’ GMFM-88 values.

Data that were analyzed in this research came from a part of the TWEC project carried out by PHU Technomex Sp. z o.o. The TWEC project concerns the realization of an IT system with a mobile application that optimizes the indications, intensity, and training loads for integrated use during technologically assisted gait education in people with CP using selected rehabilitation devices. The study included 23 patients with CP (8–24 years of age): 12 women and 11 men. The analysis used 55 data samples about patients belonging to the GMFCS 2 group that were recorded during different stages of the project (there may be from 1 to 4 samples per patient). In the project GMFM-88 scale values were used, which were determined by physicians: the results are shown in Figure 1 as a box plot, with specific values represented as blue dots (they may overlap).

### 2.4. Zebris FDM-T Treadmill Diagnostics Scheme

This section describes the Zebris FDM-T treadmill diagnostics scheme that our partners (Department of Pediatric Orthopedics and Traumatology at Poznan University of Medical Sciences and PHU Technomex Sp. z o.o.) use in the TWEC project. Assessment on the treadmill is carried out in two ways: static (standing) and dynamic (walking). The first involves standing upright, while the second allows for the analysis of individual phases of gait and the assessment of many parameters describing gait that were mentioned in Section 2.2. The static measurement is performed at least twice for 30 s with eyes open and separately with eyes closed. When performing the test with eyes open, the screen should show a neutral image with no COP (center of pressure) feedback, or the screen should be obscured. In order to be able to perform a dynamic measurement, the examined person must get used to walking on moving ground. The period of acclimatization to walking on the treadmill lasts between 5 and 6 min, during which the patient’s comfort speed is verified, trying to adjust the speed to obtain the natural locomotion speed of the ground (determined during the gait analysis in the optoelectronic system or the initial 6MWT fragment). If the patient does not feel comfortable after reaching the ground speed, the speed is gradually reduced every 30 s until the subject declares that the speed is appropriate. Then, a specific measurement is performed that lasts 60 s. After this period, the speed is gradually increased every 30 s until the maximum speed at which the patient is not afraid to move is achieved and another 60-s measurement is performed. On the treadmill, the patient walks barefoot and is allowed to hold the handrail if necessary. Patient diagnostics on the Zebris treadmill takes between 30 and 40 min.

### 2.5. Data Analysis

Many more parameters than data samples may lead to a problem with model fitting [70]. Therefore, direction of the learning process was performed. To improve the quality of the model, two types of operations were used. The first was the reduction of parameters to the ones most correlated with the target (GMFM dimensions). The second was based on an introduction of interaction features. At first, the parameters most correlated with the target were found. Then, they were multiplied by themselves and added to the original parameters. Lastly, the parameters that were most correlated with each other were removed. As a result, the remaining parameters were correlated with the target, but not with each other.

For data analysis, the Spark framework [71] was used. In particular, the MLlib library was utilized. The data were randomly divided into:

- training set—80% of the data,
- testing set—remaining 20% of the data.

Then, the Generalized Linear Regression using the Gaussian family. It allows flexible specification of a Generalized Linear Model (GLM) that can be applied to different types of predictive problems, including linear regression, Poisson regression, logistic regression, and more [71].

Finally, with the help of this tool, the following measures were calculated:

- Mean Squared Error (MSE),
- Root Mean Squared Error (RMSE),
- Mean Absolute Error (MAE),
- Mean Absolute Percentage Error (MAPE),
- Coefficient of Determination ($R^{2}$) [71].

The operations of dividing the data into training and testing were repeated 100 times. In the next section, errors are presented in two forms: plots and tables. The plots show the specific error values for each iteration as orange dots (they may overlap) and aggregated error values for all iterations in the form of a box plot. In the tables, the following symbols are used:

- min—minimal value,
- q1—first quartile,
- median—second quartile,
- q3—third quartile,
- max—maximal value,
- q3 − q1—difference between third and first quartile,
- range—difference between maximal and minimal value.

## 3. Results

In this section, experimental results concerning MSE, RMSE, MAPE, and $R^{2}$ are presented. Their description and interpretation are provided.

MSE is used to calculate the average squared difference between the observed and estimated values. RMSE is the square root of MSE. The smaller the values of these measures, the closer the model is to the actual data. The definitions used are as follows (https://spark.apache.org/docs/2.3.0/mllib-evaluation-metrics.html (accessed on 20 October 2021)):

(1) $$\mathrm{MSE}=\frac{1}{N}\sum_{i=0}^{N-1}{\left(y_{i}-\hat{y_{i}}\right)}^{2},\;\mathrm{RMSE}=\sqrt{\frac{1}{N}\sum_{i=0}^{N-1}{\left(y_{i}-\hat{y_{i}}\right)}^{2}}$$

**Figure 2:**
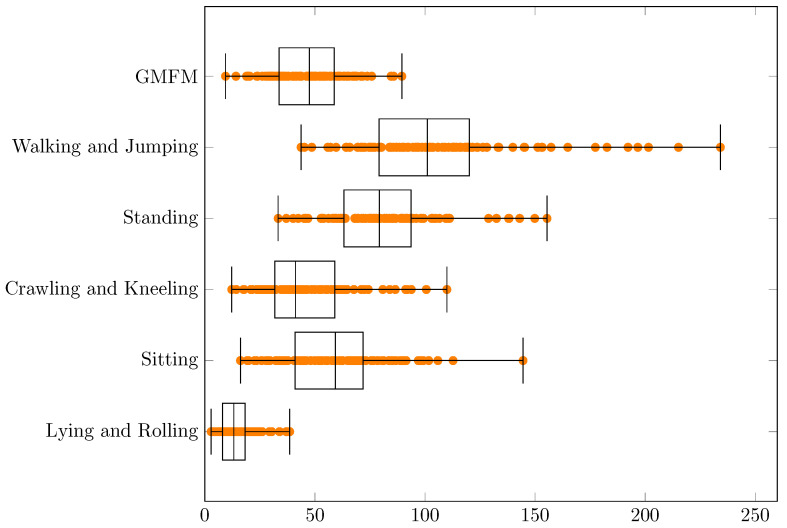
The MSE values for the conducted experiments.

**Figure 3:**
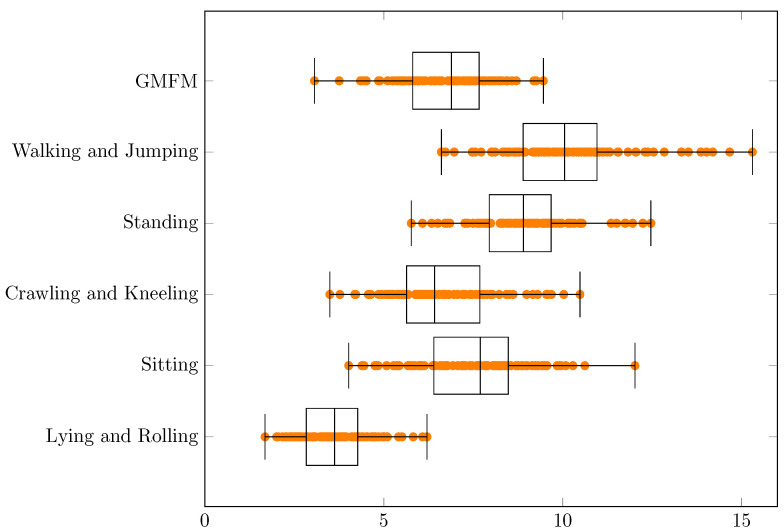
The RMSE values for the conducted experiments.

Experimental results concerning MSE and RMSE are presented in the Table 1 and Table 2 and in the Figure 2 and Figure 3, respectively.

MAE is used to calculate the average absolute difference between the observed and estimated values. MAPE is the percentage version of MAE, where the absolute difference between the observed and estimated values is referred to the first one. Similarly to MSE and RMSE, the smaller the values of these measures, the closer the model is to the actual data. The MAE penalizes large errors more than MSE. The definitions used are as follows:

(2) $$\mathrm{MAE}=\frac{1}{N}\sum_{i=0}^{N-1}\left|y_{i}-\hat{y_{i}}\right|,\;\mathrm{MAPE}=\frac{100}{N}\sum_{i=0}^{N-1}\left|\frac{y_{i}-\hat{y_{i}}}{y_{i}}\right|$$

**Figure 4:**
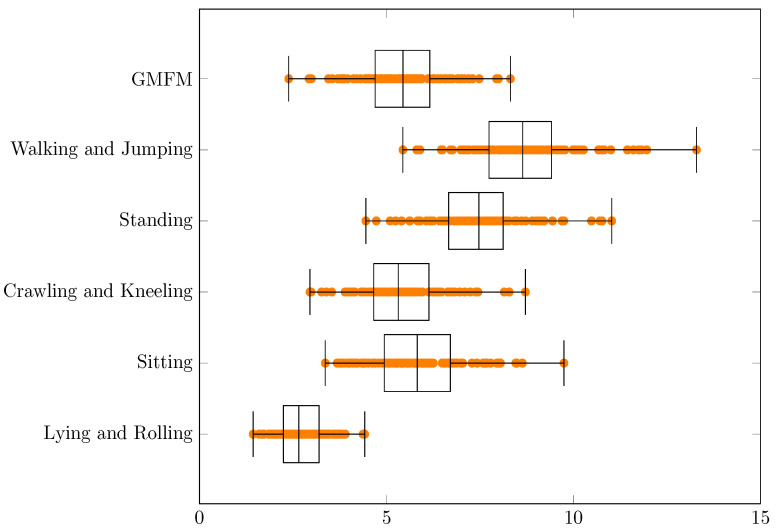
The MAE values for the conducted experiments.

**Figure 5:**
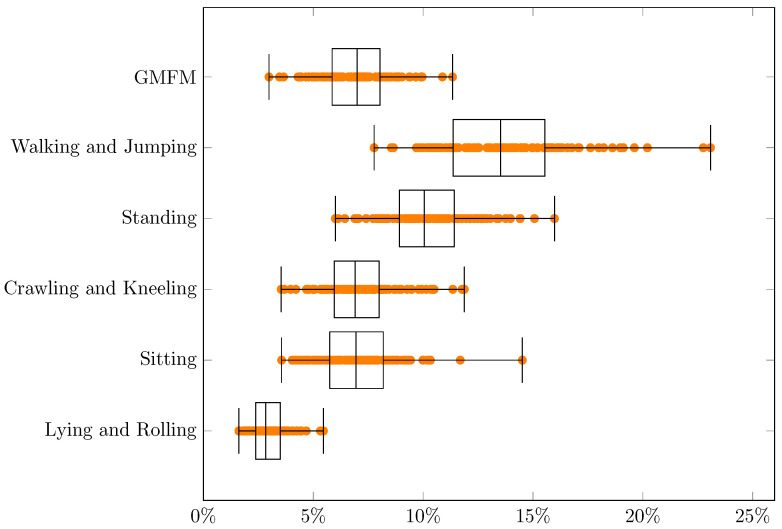
The MAPE [%] values for the conducted experiments.

Experimental results concerning MAE and MAPE are presented in the Table 3 and Table 4 and in the Figure 4 and Figure 5, respectively.

For MSE, RMSE, MAE and MAPE the smallest errors can be observed for a dimension “Lying and Rolling” and the biggest errors for “Walking and Jumping”. Significant similarities concerning MAPE between the dimensions “GMFM” (min = 3.0%, q1 = 5.9%, median = 7.0%, q3 = 8.0%, max = 11.3%, q3 − q1 = 2.2%, range = 8.4%) and “Crawling and Kneeling” (min = 3.5%, q1 = 6.0%, median = 6.9%, q3 = 8.0%, max = 11.9%, q3 − q1 = 2.0%, range = 8.3%) can be found in Table 4. This can also be noticed for various extents for MSE, RMSE and MAE (Table 1, Table 2 and Table 3, respectively). If the range (max–min) are taken into account, the smallest values always have “Lying and Rolling”, and the largest have “Walking and Jumping”. The results strongly depend on the selection of training and test data. In the best case, estimations for all dimensions can be made with a MAPE error ≤7.8%, and in the worst case ≤23.1%, which means an increase of almost 3 times. The error values in the dimensions probably follow to some extent from the actual results of the patients (Figure 1). They are also probably related to the different difficulties of the movements in the different dimensions.

$R^{2}$ is used to calculate the proportion of variance shared by the dependent variable and the independent variable(s). The higher the the value, the higher percentage of variation of dependent variable that is explained by the independent variable(s). The maximum value is 1 (100%). There are a few reason why $R^{2}$ may be negative [72], for example, the model is not good enough. According to [73] $R^{2}$ is more informative than other metrics in regression analysis evaluation. The definition used is as follows:

(3) $$R^{2}=1-\frac{\mathrm{MSE}}{\mathrm{VAR}(y)(N-1)}=1-\frac{\sum_{i=0}^{N-1}{\left(y_{i}-\hat{y_{i}}\right)}^{2}}{\sum_{i=0}^{N-1}{\left(y_{i}-\bar{y_{i}}\right)}^{2}}$$

**Figure 6:**
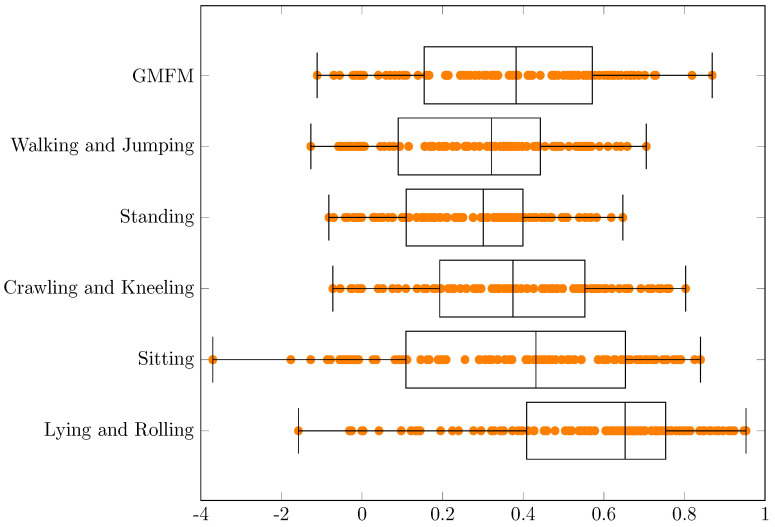
The $R^{2}$ values for the conducted experiments.

Experimental results concerning $R^{2}$ are presented in the Table 5 and in the Figure 6.

For all dimensions, at least 75% of the cases (from q1 to max) have positive values of $R^{2}$. Median varies from about 0.30 for “Standing” to about 0.75 for “Lying and Rolling”. Larger, non negative values would be desirable.

## 4. Discussion

Estimating the GMFM measure for patients with CP is a research topic that has not been extensively studied so far. Our experiments confirmed the hypothesis put forward in the introduction to the article: it is possible to estimate the value of this metric based on the Zebris FDM-T treadmill results, with the errors that were discussed in detail in the previous section. Some motor abilities (GMFM dimensions) are better estimated than others. The smallest errors can be observed for a dimension “Lying and Rolling” and the biggest errors for “Walking and Jumping”. It may be related to the varying degree of difficulty of the tasks performed within each dimension. More complex exercises may lead to higher estimation errors because the patient may make more mistakes, e.g., as a result of greater fatigue or his/her impairment. Evidence that GMFM estimation is possible can also be found in the work by Duran et al. [56], who developed a method that predicts the expected changes in the GMFM-66 measure of individual children with CP between two points in time that are 6 months apart. In their work, the LMS (lambda-mu-sigma) method was used to generate age-related reference centile curves for the GMFM-66 score.

Because the Zebris FDM-T is a widely used device in gait rehabilitation, our proposition has the potential to be widely adopted. To the best of our knowledge, the approach presented in this paper has not been tried before, so our method can be considered novel. The main disadvantage regarding the GMFM index is related to the efficiency of its administration [30,31]. That is why its abbreviated forms were developed: GMFM-66-IS and GMFM-66 B&C [32]. In [31], Duran et al. proposed yet another reduced version of the GMFM-66 index named rGMFM-66, but its administration still requires some test items to be performed by the patient, although to a limited extent. Using our proposition, a value of GMFM can be estimated immediately when data from Zebris FDM-T are accessible, which can significantly shorten the time of a patient’s examination.

Our method can be of great help for untrained therapists who are not familiar with the system of measurement. It can also be utilized by students during their didactic process. Furthermore, our proposition can be used to quickly assess the motor skills of a patient, and then, if a more thorough assessment is needed, a physician can administer the measure in a classic way.

There are many further research directions that can be investigated. It would be useful to estimate metrics with a larger sample of data from more patients. The impact of grouping patients with a similar disease type (e.g., CP type) on the estimation of the metric may also be examined. In projects that also include rehabilitation, estimation of changes in the patient’s health may be researched. Furthermore, this approach can be extended to the estimation of changes in metrics as a consequence of surgeries that patients undergo, similarly to [52,53,55,57]. In addition, estimations can be based on patient data from different medical devices that may be used individually or together.

## Figures and Tables

**Table 1:** The MSE values for the conducted experiments.

	Min	q1	Median	q3	Max	q3 − q1	Range
GMFM	9.4	33.7	47.5	58.7	89.5	25.0	80.1
Walking and Jumping	43.7	79.1	101.1	120.1	234.2	41.0	190.5
Standing	33.3	63.2	79.3	93.7	155.4	30.5	122.2
Crawling and Kneeling	12.2	31.8	41.2	59.0	109.9	27.3	97.7
Sitting	16.1	40.9	59.3	71.9	144.5	30.9	128.4
Lying and Rolling	2.8	8.0	13.1	18.2	38.5	10.2	35.7

**Table 2:** The RMSE values for the conducted experiments.

	Min	q1	Median	q3	Max	q3 − q1	Range
GMFM	3.1	5.8	6.9	7.7	9.5	1.9	6.4
Walking and Jumping	6.6	8.9	10.1	11.0	15.3	2.1	8.7
Standing	5.8	7.9	8.9	9.7	12.5	1.7	6.7
Crawling and Kneeling	3.5	5.6	6.4	7.7	10.5	2.0	7.0
Sitting	4.0	6.4	7.7	8.5	12.0	2.1	8.0
Lying and Rolling	1.7	2.8	3.6	4.3	6.2	1.4	4.5

**Table 3:** The MAE values for the conducted experiments.

	Min	q1	Median	q3	Max	q3 − q1	Range
GMFM	2.4	4.7	5.4	6.2	8.3	1.5	5.9
Walking and Jumping	5.4	7.7	8.6	9.4	13.3	1.7	7.9
Standing	4.4	6.7	7.5	8.1	11.0	1.5	6.6
Crawling and Kneeling	3.0	4.7	5.3	6.1	8.7	1.5	5.8
Sitting	3.4	4.9	5.8	6.7	9.7	1.8	6.4
Lying and Rolling	1.4	2.2	2.7	3.2	4.4	1.0	3.0

**Table 4:** The MAPE [%] values for the conducted experiments.

	Min	q1	Median	q3	Max	q3 − q1	Range
GMFM	3.0%	5.9%	7.0%	8.0%	11.3%	2.2%	8.4%
Walking and Jumping	7.8%	11.4%	13.5%	15.5%	23.1%	4.2%	15.3%
Standing	6.0%	8.9%	10.1%	11.4%	16.0%	2.5%	10.0%
Crawling and Kneeling	3.5%	6.0%	6.9%	8.0%	11.9%	2.0%	8.3%
Sitting	3.6%	5.7%	6.9%	8.2%	14.5%	2.4%	11.0%
Lying and Rolling	1.6%	2.4%	2.8%	3.5%	5.5%	1.1%	3.9%

**Table 5:** The $R^{2}$ values for the conducted experiments.

	Min	q1	Median	q3	Max	q3 − q1	Range
GMFM	−1.11	0.15	0.38	0.57	0.87	0.42	1.98
Walking and Jumping	−1.27	0.09	0.32	0.44	0.71	0.35	1.98
Standing	−0.82	0.11	0.30	0.40	0.65	0.29	1.47
Crawling and Kneeling	−0.72	0.19	0.37	0.55	0.80	0.36	1.53
Sitting	−3.70	0.11	0.43	0.65	0.84	0.54	4.54
Lying and Rolling	−1.58	0.41	0.65	0.75	0.95	0.35	2.53

## Data Availability

Data sets used and analyzed in this research are available from the corresponding author on reasonable request.

## Abbreviations

The following abbreviations are used in this manuscript:

CP	Cerebral palsy
BDS	Big Data Analytics
ML	Machine Learning
FV	Focal Vibration
CV	Computer Vision
GCSs	Gait Classification Systems
TD	Typically developing
3DIGA	Three-dimensional Instrumented Gait Analysis
GGI	Gillette Gait Index
GDI	Gait Deviation Index
GPS	Gait Profile Score
GMFM	Gross Motor Function Measure
GMFCS	Gross Motor Function Classification System
6MWT	6-m Walk Test
GOAL	Gait Outcomes Assessment List
RF	Random Forest
SVM	Support Vector Machine
CFCS	Communication Function Classification System
FTSST	Five-Times-Sit-to-Stand Test
MACS	Manual Ability Classification System
CNN	Convolutional Neural Network
RR	Ridge Regression
GCNN	Graph Convolutional Neural Network
GMAE	Gross Motor Ability Estimator
TWEC	Technologicznie Wspomagana Edukacja Chodu
COP	Center of Pressure
GLM	Generalized Linear Model
MSE	Mean Squared Error
RMSE	Root Mean Squared Error
MAE	Mean Absolute Error
MAPE	Mean Absolute Percentage Error
$R^{2}$	Coefficient of Determination
